# Supplementary material for: Liver-specific ATP-citrate lyase inhibition by bempedoic acid decreases LDL-C and attenuates atherosclerosis
Source: Nat Commun. 2016 Nov 28;7:13457. doi: 10.1038/ncomms13457 (PMC5133702; doi:10.1038/ncomms13457)
Supplement: Supplementary Information — Supplementary Figures 1-8 and Supplementary Table 1 [file ncomms13457-s1.pdf]

Supplementary Figure 1

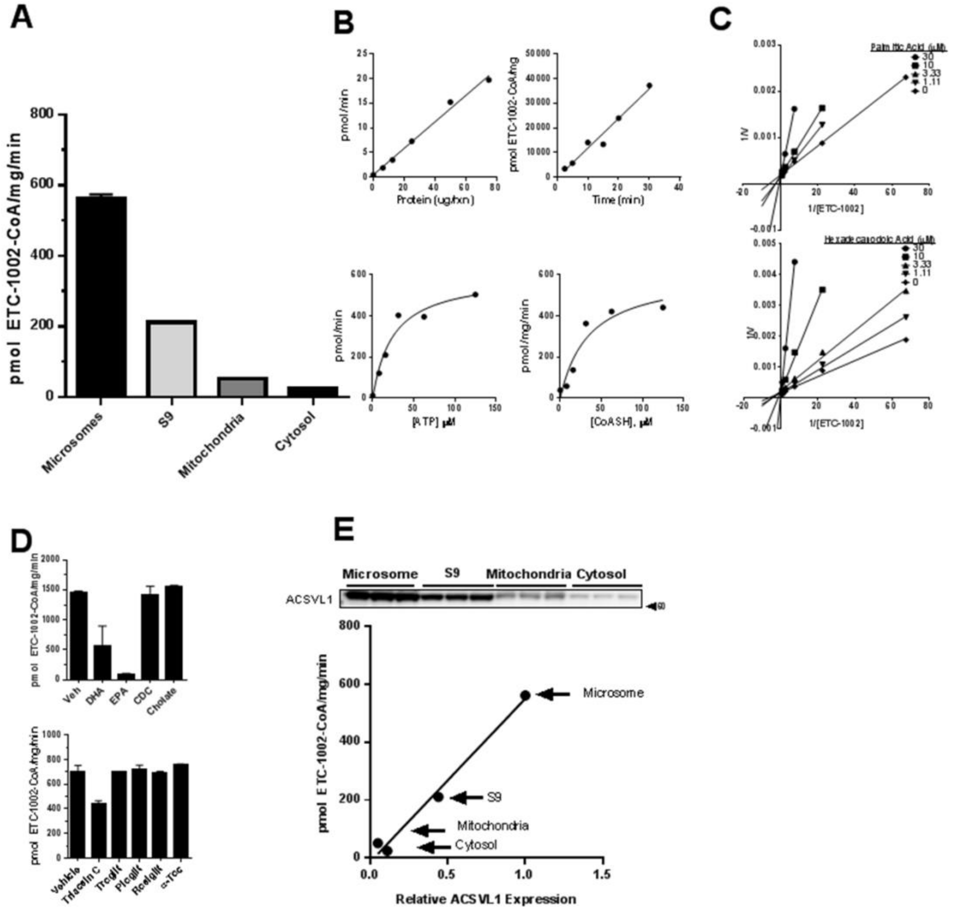

Supplementary Figure 1. Characterization of the ETC-1002-CoA synthetase. (A) Equal total protein from subcellular fractions of human liver homogenates were incubated with [^{14}C]-ETC-1002 and conversion to [^{14}C]-ETC-1002-CoA was determined. (B) ETC-1002-CoA synthetase activity was measured in human microsomes and standard assay kinetics determined. (C) [^{14}C]-ETC-1002 (10 µM) conversion to [^{14}C]-ETC-1002-CoA in human liver microsomes was determined in the presence of unlabeled substrates. Lineweaver-Burk plots represent competitive inhibition of ETC-1002-CoA synthetase activity by palmitic acid and hexadecanodoic acid against ETC-1002. (D) [^{14}C]-ETC-1002 (10 µM) conversion to [^{14}C]-ETC-1002-CoA in human liver microsomes was determined in the presence of 30 µM unlabeled competitive substrates or 10 µM inhibitors, Troglitazone (Troglit), Pioglitazone (Pioglit), Rosiglitazone (Rosiglit), and α-Tocopherol (α-Toc). (E) Relative ACSVL1 expression was determined in subcellular fractions from human liver homogenates from (A) by western blotting. Relative ACSVL1 expression was plotted against the ETC-1002-CoA synthetase activity for each respective subcellular fraction and linear regression analysis was performed. Data from liver microsome preparations (n = 50 donors pooled) are representative of mean ± SD of two independent experiments performed in duplicate (A through D), or triplicate (E).

Supplementary Figure 2

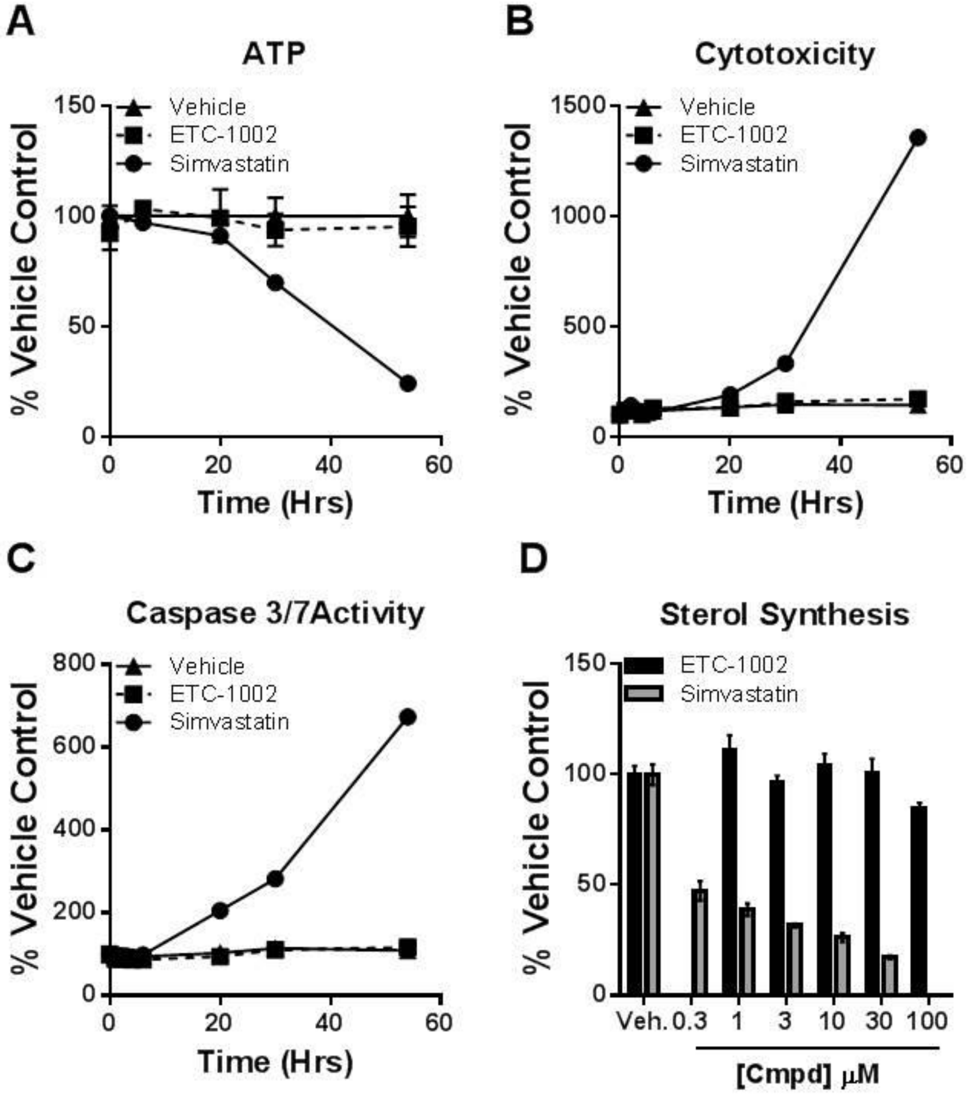

Supplementary Figure 2. Temporal effects of simvastatin and ETC-1002 on L6 cytotoxicity. L6 mytotubes were incubated with vehicle, ETC-1002 (100 μM), or simvastatin (10 μM) for the indicated times and (A) ATP, (B) cytotoxicity, and (C) Caspase 3/7 activity was determined. (D) Rates of sterol synthesis in the presence of the indicated concentrations of ETC-1002 and simvastatin were determined over a 24 hour period. (A through C) were performed in duplicate and (D) in triplicate. Data are from a single experiment and presented as mean ± SD (A through C) or percent control of mean ± SEM DPM/well (D).

Supplementary Figure 3

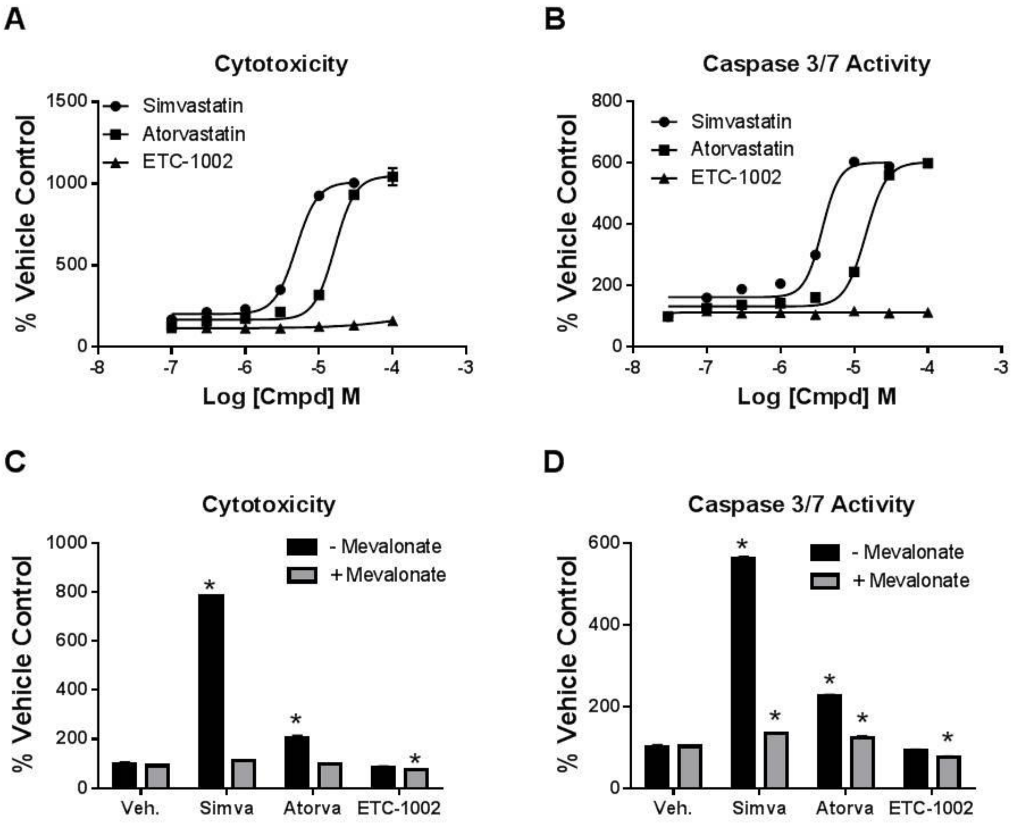

Supplementary Figure 3. Mevalonate rescues statin-induced cytotoxicity in L6 cells. L6 mytotubes were incubated with vehicle or the indicated concentrations of ETC-1002 or simvastatin for 48 hours and (A) cytotoxicity or (B) caspase 3/7 activity determined. L6 myotubes were incubated with vehicle, simvastatin (simva) (10 µM), atorvastatin (atorva) (10 µM), or ETC-1002 (100 µM) ± mevalonate (500 µM) for 48 hours and (C) cytotoxicity and (D) caspase 3/7 activity measured. Data are from a single experiment and are expressed as mean ± SEM of triplicate measures. Multiple comparisons were made using an one-way ANOVA, Bonferroni's multiple comparisons test; * $p < 0.05$.

Supplementary Figure 4

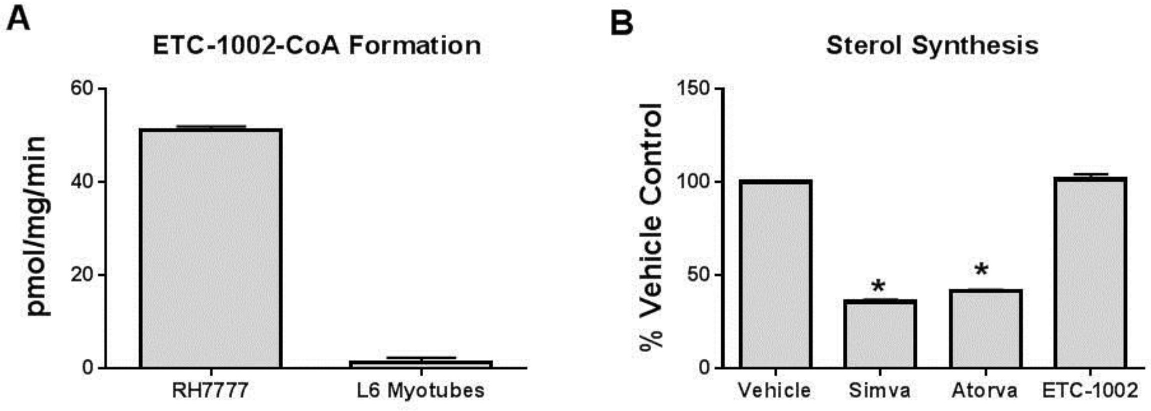

Supplementary Figure 4. ETC-1002 does not inhibit sterol synthesis in L6 myotubes. (A) McArdle (RH7777) cell and L6 myotube lysates were generated and ETC-1002-CoA synthetase activity was measured in the presence of 10 µM [14C]-ETC-1002. Data are expressed as mean ± SEM pmol ETC-1002-CoA generated per mg total protein per min; n = 3 preparations. (B) Rates of sterol synthesis were measured over 24 hours in the presence of vehicle, simvastatin (Simva) (10 µM), atorvastatin (Atorva) (10 µM), or ETC-1002 (100 µM). Data are the mean ± SEM of DPM/ mg total protein and expressed as percent control, and representative of two independent experiments performed in triplicate. Multiple comparisons were made using an one-way ANOVA, Bonferroni's multiple comparisons test; * $p < 0.05$

Supplementary Figure 5

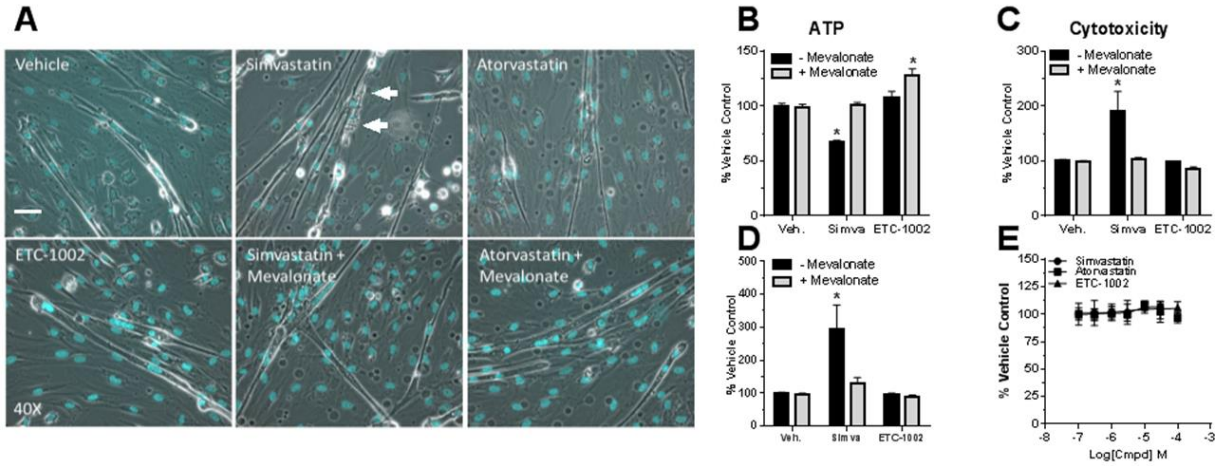

Supplementary Figure 5. Mevalonate rescues statin induced cytotoxicity in primary human myotubes. Primary human myotubes were treated with vehicle, 10 µM simvastatin (+/- 500 µM mevalonate), atorvastatin 10 µM (+/- 500 µM mevalonate), or ETC-1002 (100 µM) for 48 hours and (A) merged images of bright field and fluorescent capture of Hoechst-stained cultures (Bar = 50 µM; arrows indicate membrane blebbing), and (B) ATP, (C) cytotoxicity was measure by (GF-AFC/bis-AAF-R110 cleavage, and (D) Caspase 3,7 activity (DEVD cleavage) determined. (E) Primary human myotubes treated with 0.1 µM to 100 µM simvastatin, atorvastatin, or ETC-1002 for 12 hours and viability determined by MTT assay. Data are the mean of two independent experiments performed in triplicate. Multiple comparisons were made using an one-way ANOVA, Bonferroni's multiple comparisons test; * $p < 0.05$

Supplementary Figure 6

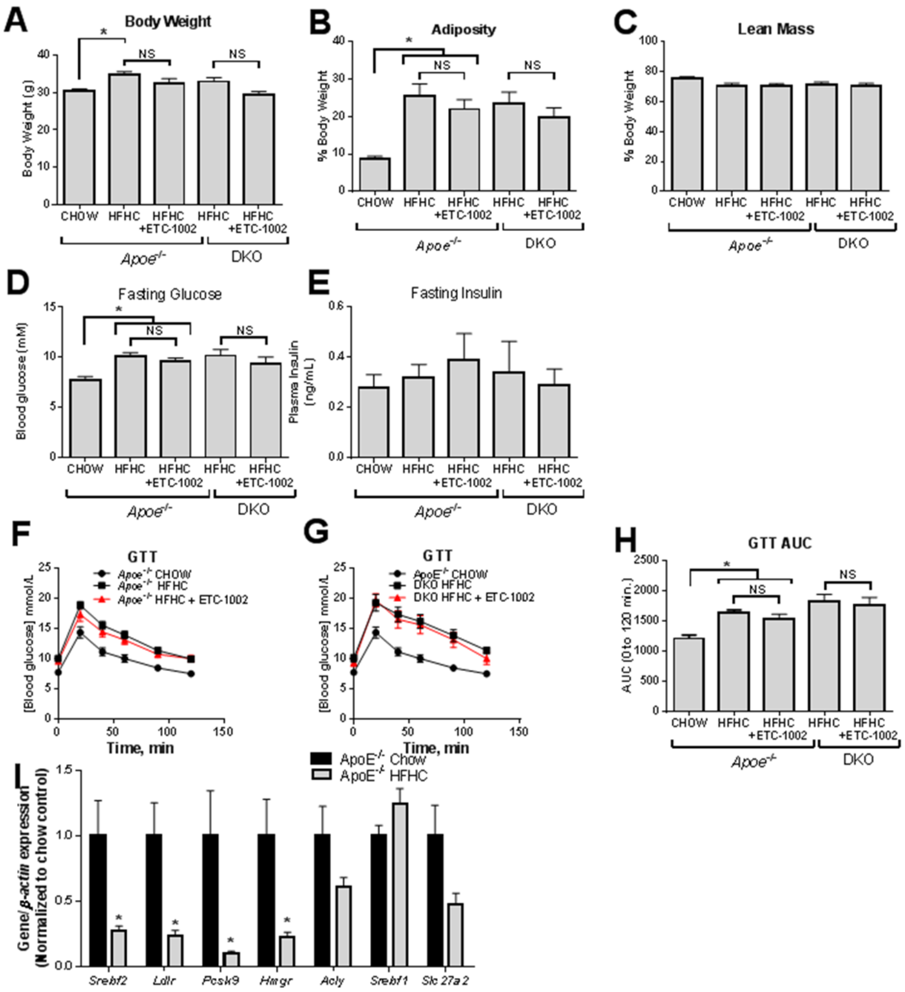

Supplementary Figure 6. Metabolic outcomes are unaffected by ETC-1002 in *Apoe*$^{-/-}$ mice. *Apoe*$^{-/-}$ and DKO mice were fed a high fat-high cholesterol (HCHF) diet for 12 weeks with or without ETC-1002 to achieve a 30 mg/kg/day dose. (A) Body mass (B) Adiposity, and (C) lean mass determined at sacrifice (n = 15 for *Apoe*$^{-/-}$, and n = 11 for control DKO, and n = 12 for ETC-1002 treated DKO). Fasting (D) glucose and (E) insulin were determined at 10 weeks on diet (glucose; n = 15 for *Apoe*$^{-/-}$, and n = 11 for control DKO, and n = 12 for ETC-1002 treated DKO; and insulin n = 6/group). Glucose tolerance test (GTT) was performed in (F) *Apoe*$^{-/-}$ and (G) DKO mice and (H) AUC$_{(0\ \text{-120 min.})}$ calculated. (I) Effects of HFHC diet on *Srebf2*, *Ldlr*, *Pcsk9*, *Hmgr*, *Acly*, *Srebf1*, and *Slc27a2* gene expression normalized to chow-fed mice was (n= 4 chow-fed and n = 5 for remaining treatment groups) determined at the end of study. Multiple comparisons were made using (I) unpaired Student's t-test or an *one-way (within *Apoe*$^{-/-}$ treatment groups) or $^{\#}$ two-way ANOVA (between *Apoe*$^{-/-}$ and DKO treatment groups); Bonferroni's multiple comparisons test; * $p < 0.05$.

Supplementary Figure 7

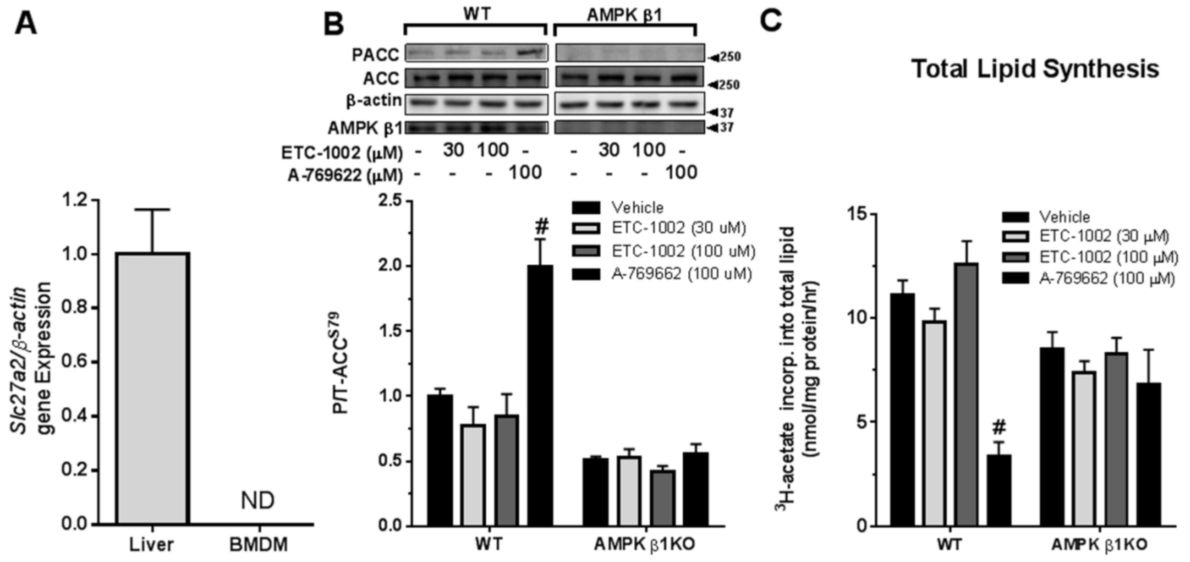

Supplementary Figure 7. ETC-1002 is inactive in bone-marrow derived macrophages. Hematopoietic stem cells were isolated from WT and β1 KO mice by taking the tibia and femur from both legs of one mouse and spinning out the bone marrow for 5 min at 4500 rpm in DMEM containing 4.5 g/L glucose, sodium pyruvate (110mg/L), 10% (v/v) fetal bovine serum, 100 IU/ml penicillin, 100 µg/ml streptomycin. The following day, the stem cells were stimulated to differentiate into BMDM under L929. (A) Following 8 days of differentiation, *Slc27a2* and β-actin expression was measured and compared to liver expression (n = 6). (B) Phosphorylation of ACC was measured in BMDM treated with Vehicle, ETC-1002 (30 and 100 µM), or A-769662 (100 µM). ETC-1002 did not increase ACC phosphorylation. (C) ETC-1002 did not lower lipogenesis in WT or β1 KO. Two-way ANOVA was used to detect statistical differences. n = 3-8 performed in triplicate measures. Data are expressed as mean ± SEM. *P < 0.05, significantly different from control.

Supplementary Figure 8

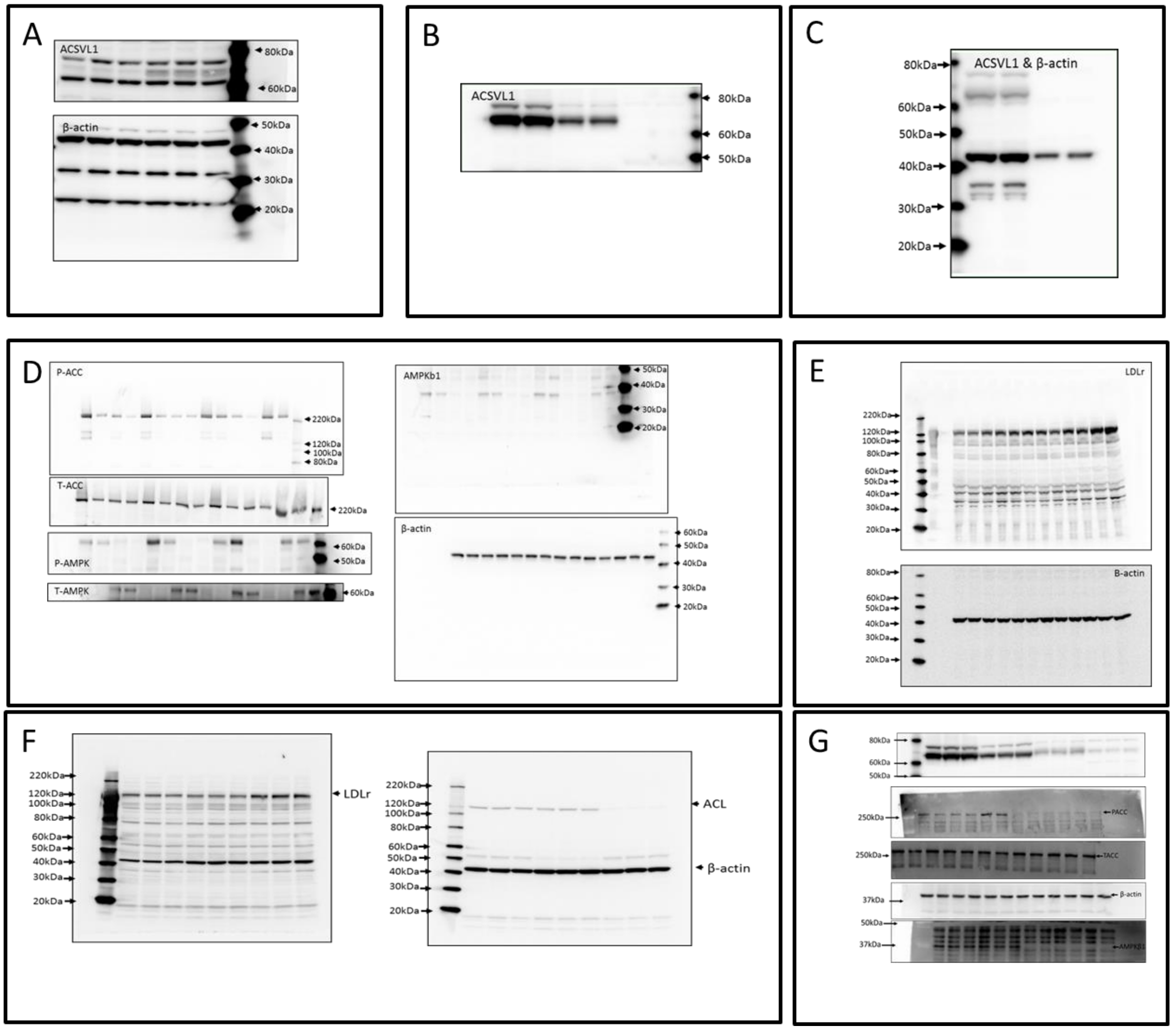

Supplementary Figure 8. Gel images. Uncropped gels are shown for figures (A) 2C, (B) 2E, (C), 2G, (D), 3A, (E) 5I, (F), 5J and (G) supplementary figures 1E and 7B.

Supplementary Table 1.

ETC-1002 Dose (mg/kg/day)	ETC-1002-CoA (ng/mg) ± SEM		
	Liver	Skeletal Muscle	Fat
10	1.4 ± 0.2	BLQ*	BLQ*
30	4.0 ± 0.7	BLQ*	BLQ*

*BLQ = Below limit of quantitation; Limit of Quantitation = 0.11 Liver, 0.22 Skeletal Muscle, and 0.01 Fat

Supplementary Table 1. ETC-1002-CoA levels in mouse liver and skeletal muscle. ETC-1002-CoA levels were measure in freeze clamped liver and skeletal muscle from high-fat fed mice receiving ETC-1002 (30 mg/kg) for 9 weeks (n = 5).
